# Primary Care-Linked Lifestyle Interventions for Type 2 Diabetes Mellitus Remission and Glucose-Lowering Medication Deintensification: A Systematic Review

**DOI:** 10.7759/cureus.114138

**Published:** 2026-08-07

**Authors:** Yasir A Agdi, Khalid I Ha‎ka‎m‎i, Abrar A H‎‎‎a‎ka‎mi, Osama A Asiri, Raghad O Alzahrani, Nesrin H Eshaq, Nura G Halawi, Nour O Alkafri, Osama A Alzahrani, Alwaleed K Alofi, Ebtihaj H Alqari

**Affiliations:** 1 Department of Family Medicine and Palliative Care, Jazan Health Cluster, Jazan, SAU; 2 College of Medicine, Jazan University, Jazan, SAU; 3 College of Medicine and Surgery, King Khaled University, Abha, SAU; 4 Faculty of Medicine, Imam Abdulrahman Bin Faisal University, Dammam, SAU; 5 College of Medicine, Ibn Sina University, Khartoum, SDN; 6 Faculty of Pharmacy, Umm Al-Qura University, Makkah, SAU; 7 College of Medicine, Hail University, Hail, SAU; 8 Department of General Practice, Al Sulaimaniyah Primary Health Care Center, East Jeddah Hospital, Jeddah First Health Cluster, Jeddah, SAU

**Keywords:** diabetes remission, glucose-lowering therapy, glycated hemoglobin, hba1c, lifestyle intervention, medication de-intensification, primary care, total diet replacement, type 2 diabetes mellitus, weight loss

## Abstract

Type 2 diabetes mellitus (T2DM) is a common chronic disease that affects millions of adults around the world. Primary care-linked lifestyle interventions offer a scalable pathway to remission. However, evidence has not been synthesized with glucose-lowering medication deintensification as a co-primary outcome or with systematic separation of randomized, proof-of-concept, and real-world implementation evidence. We conducted a systematic review and meta-analysis in accordance with the Preferred Reporting Items for Systematic Reviews and Meta-Analyses (PRISMA) 2020 guidelines. Four databases (PubMed, Scopus, Web of Science, and Cochrane Central Register of Controlled Trials (CENTRAL)) were searched in June 2026. Eligible studies included adults with T2DM enrolled in primary care-linked lifestyle interventions. Risk of bias was assessed using the Cochrane Risk of Bias 2 (RoB 2) tool and the Joanna Briggs Institute (JBI) Critical Appraisal Checklist for Quasi-Experimental Studies, and certainty of evidence was assessed using the Grading of Recommendations Assessment, Development and Evaluation (GRADE) framework. Pooled risk ratios (RRs) and mean differences were estimated using DerSimonian-Laird random-effects models. Six studies met eligibility criteria, including two primary 12-month randomized controlled trials (RCTs) and four additional proof-of-concept or implementation evidence streams. The two 12-month comparative RCTs (Diabetes Remission Clinical Trial (DiRECT) and Diabetes Intervention Acceleration through Mobilization I (DIADEM-I)) demonstrated a pooled remission RR of 7.42 (95% confidence interval (CI) 3.51-15.69; I² = 53%; moderate certainty) and medication-free status RR of 4.31 (95% CI 3.24-5.72; I² = 0%; moderate certainty). The pooled unadjusted mean difference in weight change was −8.75 kg (95% CI −10.01 to −7.48; I² = 0%), and the pooled mean difference in glycated hemoglobin change was −8.74 mmol/mol (95% CI −13.58 to −3.90; I² = 70%; low certainty). Single-arm and real-world implementation evidence showed a pooled remission proportion of 42.6% (95% CI 26.4-59.6%; I² = 95%; very low certainty), interpreted as implementation rates rather than causal treatment effects. Primary care-linked lifestyle interventions, particularly total diet replacement-based pathways, substantially increase the likelihood of T2DM remission and medication-free status at 12 months. Sustained weight loss appears to be the primary mediating mechanism. Safe implementation requires structured medication withdrawal protocols and long-term primary care follow-up.

## Introduction and background

Type 2 diabetes mellitus (T2DM) is one of the most consequential chronic diseases worldwide. An estimated 589 million adults aged 20-79 years were living with diabetes in 2024, representing 11.1% of the global adult population, with prevalence projected to increase substantially over the coming decades [1,2]. T2DM accounts for approximately 90% of diabetes cases and is associated with major morbidity, mortality, healthcare expenditure, and complications, including nephropathy, retinopathy, peripheral neuropathy, myocardial infarction, and stroke [3-5]. Management has traditionally focused on lifelong glucose-lowering therapy; however, the concept of T2DM as an inevitably progressive and irreversible condition has been challenged by evidence demonstrating that remission is achievable in a proportion of individuals [1,4].

Remission is defined as sustained glycemic levels below the diabetes threshold without glucose-lowering medication, with a glycated hemoglobin (HbA1c) level below 6.5% (48 mmol/mol) measured at least three months after withdrawal of pharmacotherapy [2,6]. This represents a shift from long-term disease control toward remission-oriented care, with potential benefits including reduced medication burden, improved quality of life, and lower healthcare costs [2,3]. The biological basis for remission is supported by the twin cycle hypothesis, which proposes that chronic excess energy intake promotes fat accumulation in the liver and pancreas, impairing insulin sensitivity and beta-cell function. These processes may be reversible through substantial weight loss, with reductions in intra-organ fat associated with improved metabolic function and restoration of insulin secretion in individuals with preserved beta-cell capacity [5-8].

Structured lifestyle interventions, particularly total diet replacement (TDR) programs involving very low-calorie diets followed by food reintroduction and weight maintenance support, have demonstrated substantial potential to induce remission [1,4]. Other dietary approaches, including lower-carbohydrate strategies, have also shown benefit, although outcomes vary according to intervention intensity, magnitude of weight loss, diabetes duration, and medication withdrawal protocols [3,5,7]. Primary care is a critical setting for delivering remission interventions because it provides access to large populations of people with T2DM, continuity of care, prescribing capability, and infrastructure for safe medication management and long-term follow-up. Evidence from primary care-delivered programs suggests that structured lifestyle pathways can be implemented beyond specialist research settings [3-7].

Despite increasing evidence supporting lifestyle-induced remission, important gaps remain. Previous reviews have examined specific dietary approaches or broader non-surgical interventions but have not focused specifically on primary care-linked lifestyle interventions [2-6]. Furthermore, glucose-lowering medication deintensification has rarely been evaluated as a co-primary outcome alongside remission, despite medication-free status being clinically important and integral to remission definitions. Existing reviews have also not consistently distinguished among evidence from randomized controlled trials (RCTs), proof-of-concept studies, and real-world implementation studies, which represent distinct levels of clinical and operational evidence [3-9]. Therefore, this systematic review and meta-analysis aimed to evaluate the effectiveness of primary care-linked lifestyle interventions for achieving T2DM remission and glucose-lowering medication deintensification, while separately synthesizing comparative RCT evidence, proof-of-concept evidence, and real-world implementation evidence.

## Review

Methods

Protocol

This systematic review and meta-analysis was conducted and reported according to the Preferred Reporting Items for Systematic Reviews and Meta-Analyses (PRISMA) 2020 guidelines [10]. A protocol was developed a priori, specifying the review question, eligibility criteria, search strategy, data extraction approach, and analysis plan before study selection and synthesis.

Review Question and Eligibility Criteria

The review assessed the effectiveness of primary care-linked lifestyle interventions in adults with T2DM for achieving diabetes remission and reducing or discontinuing glucose-lowering medication compared with usual care, delayed intervention, or baseline status.

Eligible participants were adults aged 18 years or older with confirmed T2DM recruited from primary care, community health, general practice, or primary care-linked settings. Studies involving type 1 diabetes, gestational diabetes, or prediabetes were excluded.

Eligible interventions included primary care-linked lifestyle programs targeting glycemic improvement, remission, or medication reduction. These included formula-based TDR, low-energy or very-low-energy dietary programs, lower-carbohydrate dietary approaches, and multicomponent interventions incorporating diet, physical activity, and behavioral support with primary care involvement in medication management or follow-up.

Eligible comparators included usual medical care, guideline-based diabetes care, diabetes education, routine lifestyle advice, delayed intervention, waiting-list control, baseline status in single-arm studies, or no comparator in service evaluations.

The primary outcomes were T2DM remission, defined as HbA1c below 6.5% (48 mmol/mol) without glucose-lowering medication for a specified minimum duration, and glucose-lowering medication deintensification. Secondary outcomes included weight change, HbA1c change, normoglycemia, cardiovascular risk factors, quality of life, physical activity, intervention acceptability, completion rates, and serious adverse events.

Search Strategy and Information Sources

A comprehensive search was conducted in PubMed, Scopus, Web of Science, and the Cochrane Central Register of Controlled Trials (CENTRAL) in June 2026. Search terms covered three concepts: T2DM; lifestyle and dietary interventions; and remission or medication deintensification. Controlled vocabulary terms and free-text keywords were combined using Boolean operators and adapted for each database.

No date restriction was applied. Searches were not restricted by language; however, only English-language publications were eligible for inclusion. Primary care linkage was assessed during screening rather than included as a search term. Reference lists of included studies and relevant reviews were manually searched for additional eligible studies.

Study Selection

Records were imported into reference management software, and duplicates were removed. Titles and abstracts were screened first, followed by full-text assessment of potentially eligible studies. Reasons for exclusion were recorded, and the selection process was summarized using a PRISMA 2020 flow diagram.

Data Extraction

Data were extracted using a standardized form covering study characteristics, participant demographics, intervention and comparator details, and outcome data. Studies reporting data only as medians or interquartile ranges were synthesized narratively rather than included in pooled analyses.

Risk of Bias Assessment

Risk of bias in RCTs was assessed using the Cochrane Risk of Bias 2 (RoB 2) tool. The assessment considered bias arising from randomization, deviations from intended interventions, missing outcome data, outcome measurement, and selective reporting [11]. Overall judgments were classified as low risk, some concerns, or high risk.

Non-randomized and single-arm studies were assessed using the Joanna Briggs Institute (JBI) Critical Appraisal Checklist for Quasi-Experimental Studies. Domains included participant comparability, intervention consistency, presence of controls, follow-up completeness, outcome measurement, and appropriateness of statistical analysis. Lack of a control group in single-arm studies was considered a design feature rather than a quality failure [12].

Quantitative Synthesis

Risk ratios (RRs) were used for binary outcomes, including remission and medication-free status. Mean differences (MDs) were used for continuous outcomes, including weight and HbA1c change. Pooled estimates were calculated using DerSimonian-Laird random-effects models. Because only two trials contributed to the primary meta-analysis, heterogeneity estimates were interpreted cautiously. Publication bias was not assessed using funnel plots because fewer than 10 studies were available for each pooled analysis. Short-term comparative evidence was analyzed separately because outcomes were assessed after the intervention phase rather than at 12 months. Single-arm remission proportions from implementation and observational studies were synthesized separately and interpreted as implementation rates rather than causal treatment effects. Individual proportions were calculated with Wilson 95% confidence intervals (CIs), and pooled proportions were estimated using Freeman-Tukey double arcsine transformation with a random-effects model [13].

Heterogeneity and Sensitivity Analyses

Clinical and methodological heterogeneity were assessed according to study design, intervention characteristics, delivery setting, participant characteristics, baseline measures, medication use, follow-up duration, remission definitions, medication-free duration criteria, and outcome ascertainment. Formal subgroup analyses and meta-regression were not performed because of the limited number of comparative studies.

Narrative Synthesis

A structured narrative synthesis was performed across all included studies, covering remission, glucose-lowering medication deintensification, weight change, HbA1c outcomes, safety, intervention acceptability, completion, and implementation within primary care. Evidence was considered separately for 12-month comparative RCTs, short-term proof-of-concept studies, and single-arm or real-world implementation studies.

Certainty of Evidence Assessment

Certainty of evidence was assessed using the Grading of Recommendations Assessment, Development and Evaluation (GRADE) framework for remission, medication-free status, weight change, HbA1c change, and serious adverse events [14]. Certainty was assessed separately for randomized evidence and observational or single-arm evidence. Evidence was downgraded for risk of bias, inconsistency, indirectness, imprecision, and publication bias where appropriate.

Results

Study Selection

**Figure 1:**
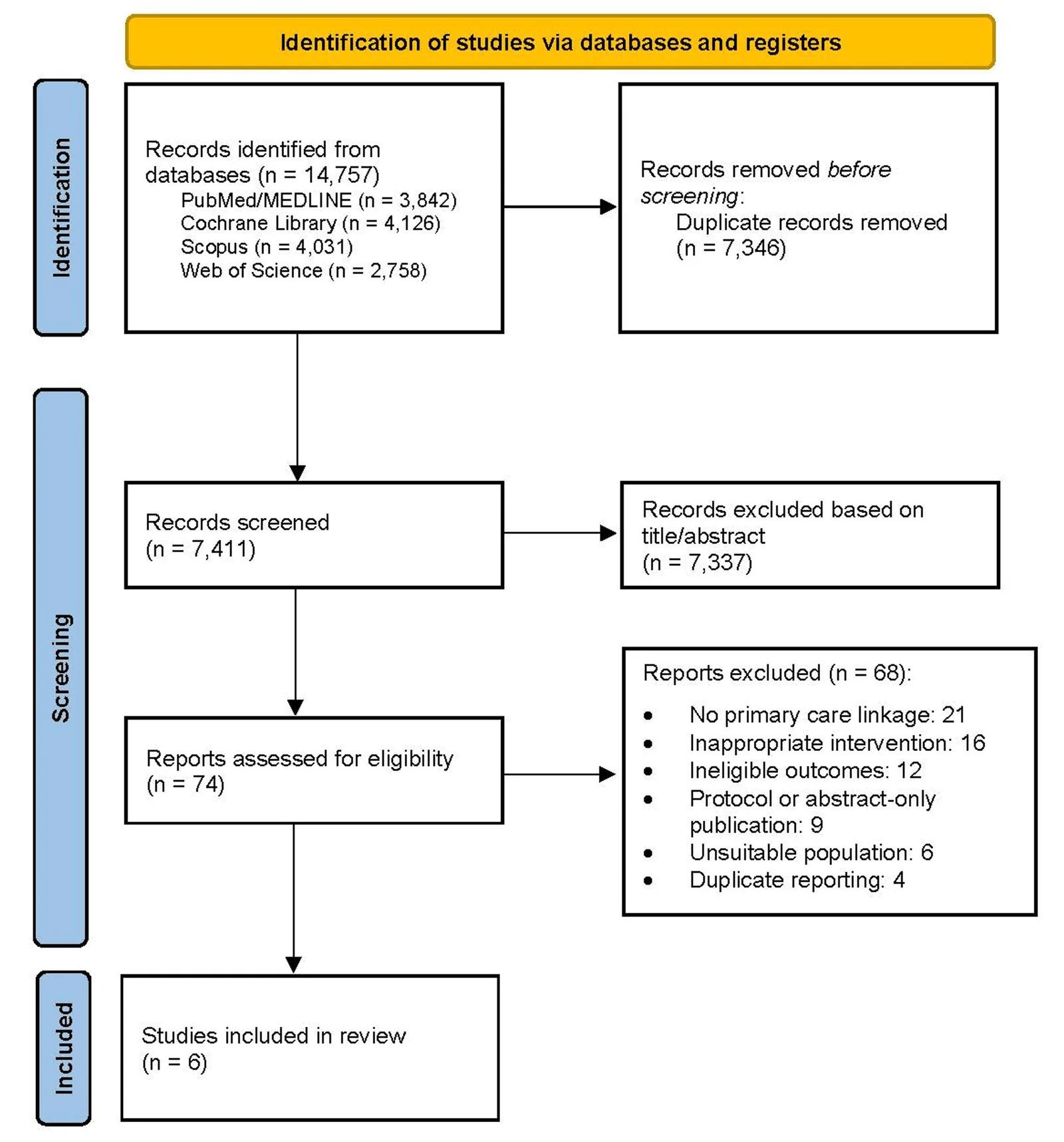
PRISMA 2020 flow diagram depicting the study selection process for the systematic review PRISMA 2020 flow diagram detailing the study selection process for this systematic review. After identifying records through database searching and other sources, duplicates were removed, and the remaining records were screened. Full-text articles were assessed for eligibility, with reasons for exclusion documented. Studies meeting the inclusion criteria were included in the final synthesis. PRISMA: Preferred Reporting Items for Systematic Reviews and Meta-Analyses

The search identified 14,757 records from four databases (PubMed, Scopus, Web of Science, and Cochrane/CENTRAL). After removal of 7,346 duplicates, 7,411 records were screened, and 74 full-text articles were assessed for eligibility. Sixty-eight articles were excluded due to lack of primary care linkage (n = 21), inappropriate intervention (n = 16), ineligible outcomes (n = 12), protocol or abstract-only publication (n = 9), unsuitable population (n = 6), or duplicate reporting (n = 4). Six studies met the inclusion criteria and were included in the qualitative synthesis (Figure 1). All six studies contributed to quantitative analyses, while two 12-month comparative RCTs, DiRECT and DIADEM-I, contributed to the main meta-analysis [1,2].

The included studies were analyzed within three evidence streams: two 12-month comparative RCTs (Diabetes Remission Clinical Trial (DiRECT) and Diabetes Intervention Acceleration Through Mobilization I (DIADEM-I)), one short-term proof-of-concept RCT (STANDby), and four single-arm or real-world implementation studies (DiRECT-Aus, NHS Type 2 Diabetes Remission (NHS T2DR), Norwood GP, and the STANDby observational extension). STANDby contributed to both randomized and observational analyses; therefore, evidence streams represent analytical categories rather than unique study counts (Table 1) [3].

**Table 1:** Baseline characteristics of included studies Baseline demographic and clinical characteristics of participants included in the systematic review. Values are presented as reported in the original studies. Where applicable, intervention (I) and control (C) group values are shown separately. BMI: body mass index, HbA1c: glycated hemoglobin, GLM: glucose-lowering medication, N: number of participants analyzed, RCT: randomized controlled trial, UK: United Kingdom, DiRECT: Diabetes Remission Clinical Trial, DIADEM-I: Diabetes Intervention Acceleration Through Mobilization I, NHS T2DR: National Health Service Type 2 Diabetes Remission, DiRECT-Aus: Diabetes Remission Clinical Trial-Australia

Study ID	Country	Design	N analysed	Mean age	Diabetes duration	Baseline BMI	Baseline HbA1c	GLM use
Lean et al., 2018 [1] (DiRECT)	UK	Open-label cluster-RCT; 49 general practices (23 intervention, 26 control)	149/149	52.9 (I) vs 55.9 (C) years	3.0 years (both groups)	35.1 vs 34.2 kg/m²	60 vs 58 mmol/mol	74.5% vs 77.2%
Taheri et al., 2020 [2] (DIADEM-I)	Qatar	Open-label individually randomized parallel-group RCT; primary and community health centers	70/77	41.9 (I) vs 42.3 (C) years	21.9 vs 20.5 months	35.0 vs 34.8 kg/m²	52.5 mmol/mol (both groups)	91.4% vs 88.3%
Sattar et al., 2023 [3] (STANDby, RCT phase)	UK	Open-label proof-of-concept RCT; primary care and social media recruitment; South Asian adults	46369	45.8 years (overall)	1.9 years (overall)	32.1 kg/m²	60.4 mmol/mol	56% (overall)
Sattar et al., 2023 [3] (STANDby, observational extension)	UK	Observational extension; all TDR starters pooled	23	45.8 years	1.9 years	32.1 kg/m²	60.4 mmol/mol	56%
Hocking et al., 2024 [4] (DiRECT-Aus)	Australia	Single-arm open-label trial; 25 general practices (New South Wales)	155 (intention-to-treat)	52.5 years	2.8 years	37.7 kg/m²	54 mmol/mol	87.7%
Valabhji et al., 2024 [5] (NHS T2DR)	England, UK	National prospective service evaluation; NHS general practice referral; six commercial providers	1,710 (full-opportunity)	~50 years	47% diagnosed <1 year	38.0 kg/m²	58.5 mmol/mol	66%
Unwin et al., 2020 [6] (Norwood GP)	England, UK	General practice service evaluation; retrospective routine data; single practice; mean 23 months	128	63 years (median)	Not reported separately	Median 99.7 kg	Median 65.5 mmol/mol	42% (54/128)

Study Characteristics

All six studies included adults with T2DM and were linked to primary care or general practice. The studies evaluated intensive dietary weight-loss interventions across different healthcare settings and populations. DiRECT was a cluster RCT conducted in UK primary care practices, whereas DIADEM-I was an individually randomized trial conducted in Qatar among a younger Middle Eastern and North African population [1,2]. STANDby evaluated the feasibility of TDR among South Asian adults in UK primary care (Table 2) [3].

**Table 2:** Characteristics of lifestyle interventions and primary care delivery models Summary of intervention characteristics, including dietary approach, prescribed energy intake, intervention duration, delivery personnel, medication withdrawal strategies, and degree of primary care involvement. TDR: total diet replacement, GP: general practitioner, BMI: body mass index, GLM: glucose-lowering medication, DiRECT: Diabetes Remission Clinical Trial, DIADEM-I: Diabetes Intervention Acceleration Through Mobilization I, NHS T2DR: National Health Service Type 2 Diabetes Remission, DiRECT-Aus: Diabetes Remission Clinical Trial-Australia

Study ID	Diet type	Energy prescription	Intervention duration	Delivery provider	Medication withdrawal protocol	Primary care linkage
Lean et al., 2018 [1] (DiRECT)	Formula TDR (Counterweight-Plus)	825-853 kcal/day	3 months (up to 5 months)	Primary care nurses/dietitians; monthly GP review	Oral glucose-lowering and antihypertensive medications stopped at TDR initiation; reintroduced per protocol	Conducted in 49 GP practices; GP retained clinical responsibility
Taheri et al., 2020 [2] (DIADEM-I)	Formula TDR (Cambridge Weight Plan)	800-820 kcal/day	12 weeks TDR, 12 weeks food reintroduction, 6 months maintenance	Dietitians, physicians, personal trainers	All glucose-lowering medications stopped at intervention initiation	Primary/community health centers with GP record screening
Sattar et al., 2023 [3] (STANDby)	Formula TDR (Counterweight-Plus)	825-853 kcal/day	3-5 months TDR, 6-8 weeks food reintroduction	Research dietitians; GP handover	Oral glucose-lowering and antihypertensive medications stopped at TDR initiation; GP-led reintroduction if required	GP recruitment with GP follow-up
Hocking et al., 2024 [4] (DiRECT-Aus)	Formula TDR (Optifast)	<800 kcal/day (BMI ≤40); <950 kcal/day (BMI >40)	13 weeks TDR, 8 weeks reintroduction, 31 weeks maintenance	Dietitians; monthly GP review	All glucose-lowering medications stopped at TDR initiation; reintroduced per protocol	Conducted in 25 GP practices; GP retained clinical responsibility
Valabhji et al., 2024 [5] (NHS T2DR)	Formula TDR	~800-900 kcal/day	12 weeks TDR, 4-6 weeks reintroduction, monthly maintenance	Commercial providers (health coaches/dietitians)	Medication-free status incorporated into remission protocol; GP retained clinical responsibility	GP referral pathway
Unwin et al., 2020 [6] (Norwood GP)	Lower-carbohydrate diet (no TDR)	No calorie target	Ongoing (mean 23 months)	GPs and practice nurses	Medication adjustment supervised by GP/practice nurse	Delivered entirely within a GP practice

Real-world studies included DiRECT-Aus in Australian general practices, NHS T2DR in England, the Norwood GP service evaluation, and the STANDby observational extension. These studies provided evidence on implementation, scalability, and delivery of remission-focused dietary interventions in routine care settings [4-6].

Intervention Characteristics

Five studies used formula-based TDR, while Norwood GP used a lower-carbohydrate dietary approach delivered through routine primary care consultations [6]. TDR interventions generally consisted of an initial low-energy formula phase followed by food reintroduction and weight-maintenance support. Glucose-lowering medications were typically discontinued at TDR initiation and reintroduced according to predefined glycemic criteria. In NHS T2DR, medication-free status was incorporated into the remission definition, with ongoing clinical responsibility retained by general practice [1-5].

Risk of Bias and Quality Assessment

The two main RCTs, DiRECT and DIADEM-I, were judged to have some concerns regarding risk of bias, mainly because of open-label intervention delivery and design limitations [1,2]. STANDby was considered high risk of bias due to substantial attrition, short follow-up, and its small proof-of-concept design [3].

The non-randomized studies were assessed as being of low-to-moderate or moderate quality. Key limitations included absence of comparator groups, incomplete follow-up, retrospective designs, small sample sizes, and potential selection bias. These studies were therefore interpreted as evidence of feasibility and implementation rather than causal effectiveness (Tables 3-4) [4-6].

**Table 3:** Risk of bias assessment of RCTs using RoB 2 Risk of bias assessment for randomized studies included in the systematic review using the RoB 2 framework [11]. Domains assessed included bias arising from the randomization process (D1), bias due to deviations from intended interventions (D2), bias due to missing outcome data (D3), bias in measurement of outcomes (D4), and bias in selection of the reported result (D5). Overall risk of bias judgments were classified as low risk, some concerns, or high risk. D1b refers specifically to assessment of timing of recruitment and identification in cluster-randomized trials. N/A: not applicable, RCTs: randomized controlled trials, RoB 2: Cochrane Risk of Bias 2, DiRECT: Diabetes Remission Clinical Trial, DIADEM-I: Diabetes Intervention Acceleration Through Mobilization I

Study ID	D1: randomisation	D1b: cluster timing	D2: deviations from intended interventions	D3: missing data	D4: outcome measurement	D5: selection of reported result	Overall risk of bias
Lean et al., 2018 [1] (DiRECT)	Low risk	Some concerns	Some concerns	Low risk	Low risk	Low risk	Some concerns
Taheri et al., 2020 [2] (DIADEM-I)	Low risk	N/A	Some concerns	Some concerns	Low risk	Low risk	Some concerns
Sattar et al., 2023 [3] (STANDby)	Some concerns	N/A	Some concerns	High risk	Some concerns	Some concerns	High risk

**Table 4:** Quality assessment of non-randomized and single-arm studies using the JBI Critical Appraisal Checklist for Quasi-Experimental Studies Methodological quality assessment of non-randomized and single-arm studies using the JBI Critical Appraisal Checklist for Quasi-Experimental Studies [12]. Domains evaluated included cause-effect relationships, participant similarity, similarity of care, presence of control groups, outcome measurement procedures, follow-up completeness, measurement reliability, and appropriateness of statistical analysis. Overall quality ratings reflect the authors’ assessment based on checklist findings. JBI: Joanna Briggs Institute, N/A: not applicable, NHS T2DR: National Health Service Type 2 Diabetes Remission, DiRECT-Aus: Diabetes Remission Clinical Trial-Australia

Study ID	JBI 1: cause and effect	JBI 2: similar participants	JBI 3: similar care	JBI 4: control group	JBI 5: multiple measurements	JBI 6: complete follow-up	JBI 7: same outcome measurement	JBI 8: reliable measurement	JBI 9: appropriate analysis	Overall quality
Sattar et al., 2023 [3] (STANDby observational)	Yes	N/A	N/A	No	Partial	Partial	Partial	Partial	Partial	Low-to-moderate quality
Hocking et al., 2024 [4] (DiRECT-Aus)	Yes	N/A	N/A	No	Yes	Partial	Yes	Yes	Yes	Moderate quality
Valabhji et al., 2024 [5] (NHS T2DR)	Yes	N/A	N/A	No	Partial	No	Partial	Partial	Partial	Moderate quality (moderate-to-serious risk of bias)
Unwin et al., 2020 [6] (Norwood GP)	Yes	N/A	N/A	No	Partial	Partial	Yes	Yes	Partial	Moderate quality

Meta-Analysis Results

**Figure 2:**
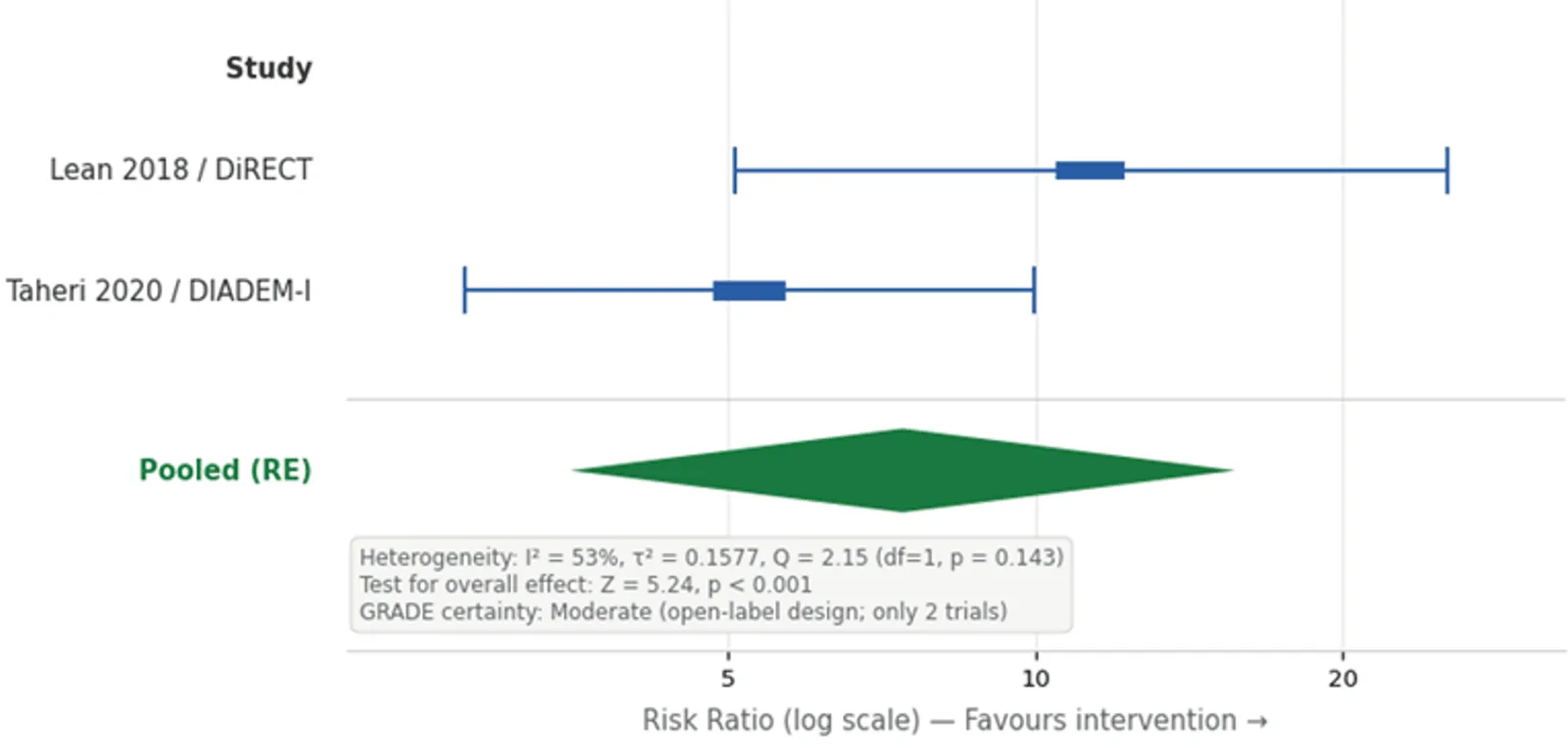
Forest plot of T2DM remission at 12 months RRs for remission comparing primary care-linked lifestyle interventions with usual care were pooled using a DerSimonian-Laird random-effects model [7]. Data were derived from Lean et al. (DiRECT) and Taheri et al. (DIADEM-I) [1,2]. The pooled effect estimate showed a higher likelihood of remission with lifestyle intervention (RR 7.42, 95% CI 3.51-15.69), with moderate heterogeneity (I² = 53.4%). Publication bias was not assessed because fewer than 10 studies were available. T2DM: type 2 diabetes mellitus, RRs: risk ratios, CI: confidence interval, DiRECT: Diabetes Remission Clinical Trial, DIADEM-I: Diabetes Intervention Acceleration Through Mobilization I

**Figure 3:**
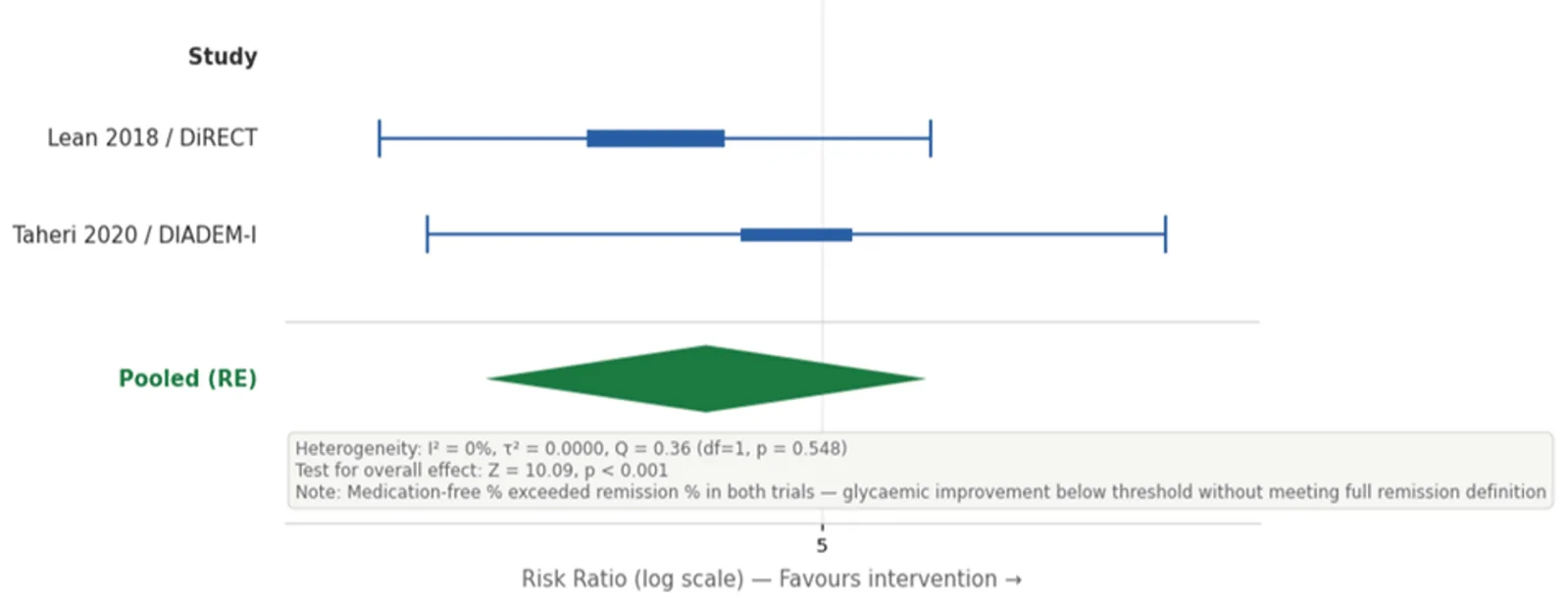
Forest plot of medication-free status at 12 months RRs for glucose-lowering medication-free status comparing primary care-linked lifestyle interventions with usual care were pooled using a DerSimonian-Laird random-effects model [7]. Data were derived from Lean et al. (DiRECT) and Taheri et al. (DIADEM-I) [1,2]. The pooled effect estimate demonstrated a higher probability of medication-free status following lifestyle intervention (RR 4.31, 95% CI 3.24-5.72), with no observed heterogeneity (I² = 0%). RRs: risk ratios, CI: confidence interval, DiRECT: Diabetes Remission Clinical Trial, DIADEM-I: Diabetes Intervention Acceleration Through Mobilization I

**Figure 4:**
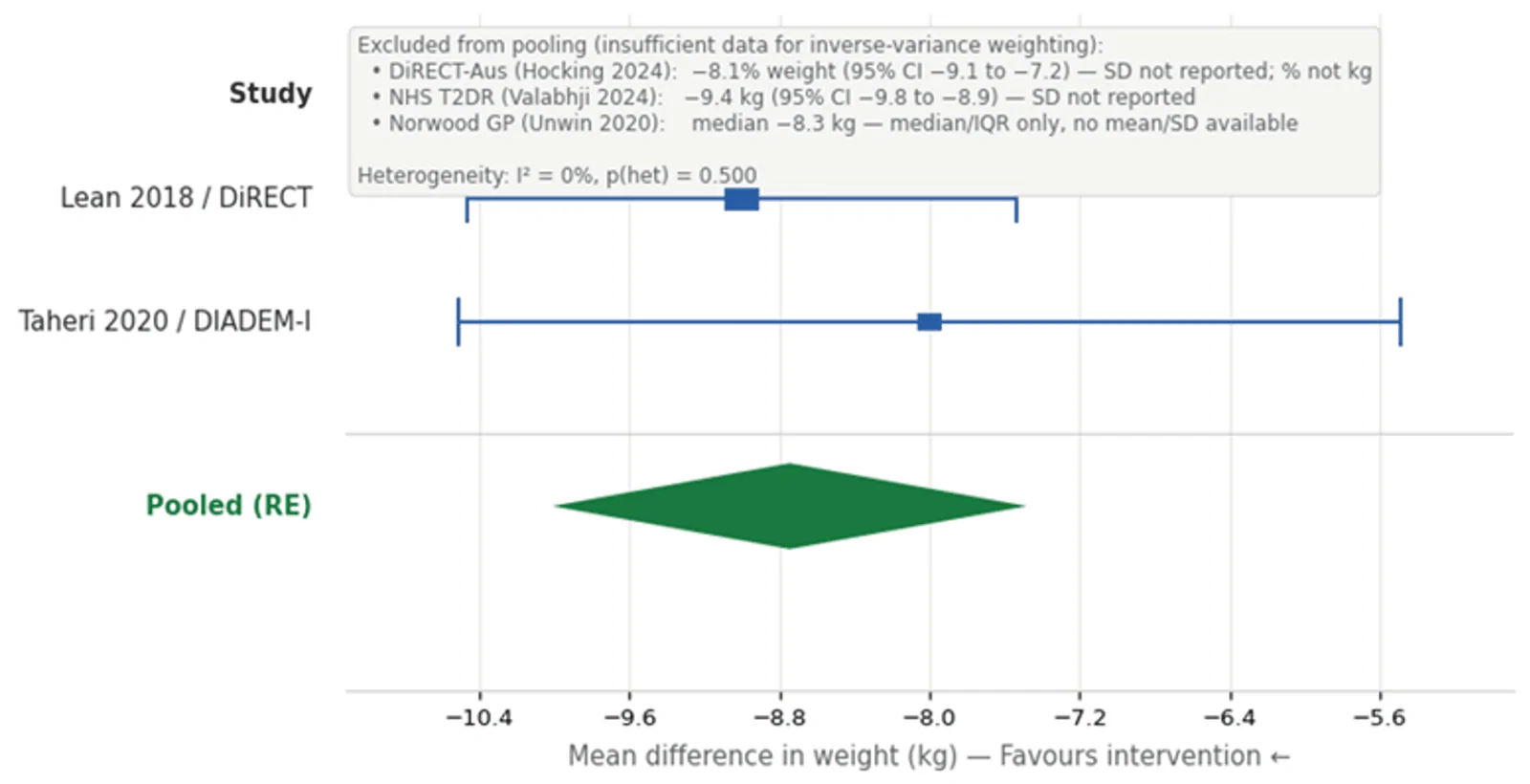
Forest plot of weight change at 12 months Unadjusted between-group MDs in weight change were pooled using a DerSimonian–Laird random-effects model [7]. Data were derived from Lean et al. (DiRECT) and Taheri et al. (DIADEM-I) [1,2]. Lifestyle interventions resulted in greater weight reduction compared with usual care (pooled MD −8.75 kg, 95% CI −10.01 to −7.48), with no observed heterogeneity (I² = 0%). MD: mean difference, CI: confidence interval, DiRECT: Diabetes Remission Clinical Trial, DIADEM-I: Diabetes Intervention Acceleration Through Mobilization I

**Figure 5:**
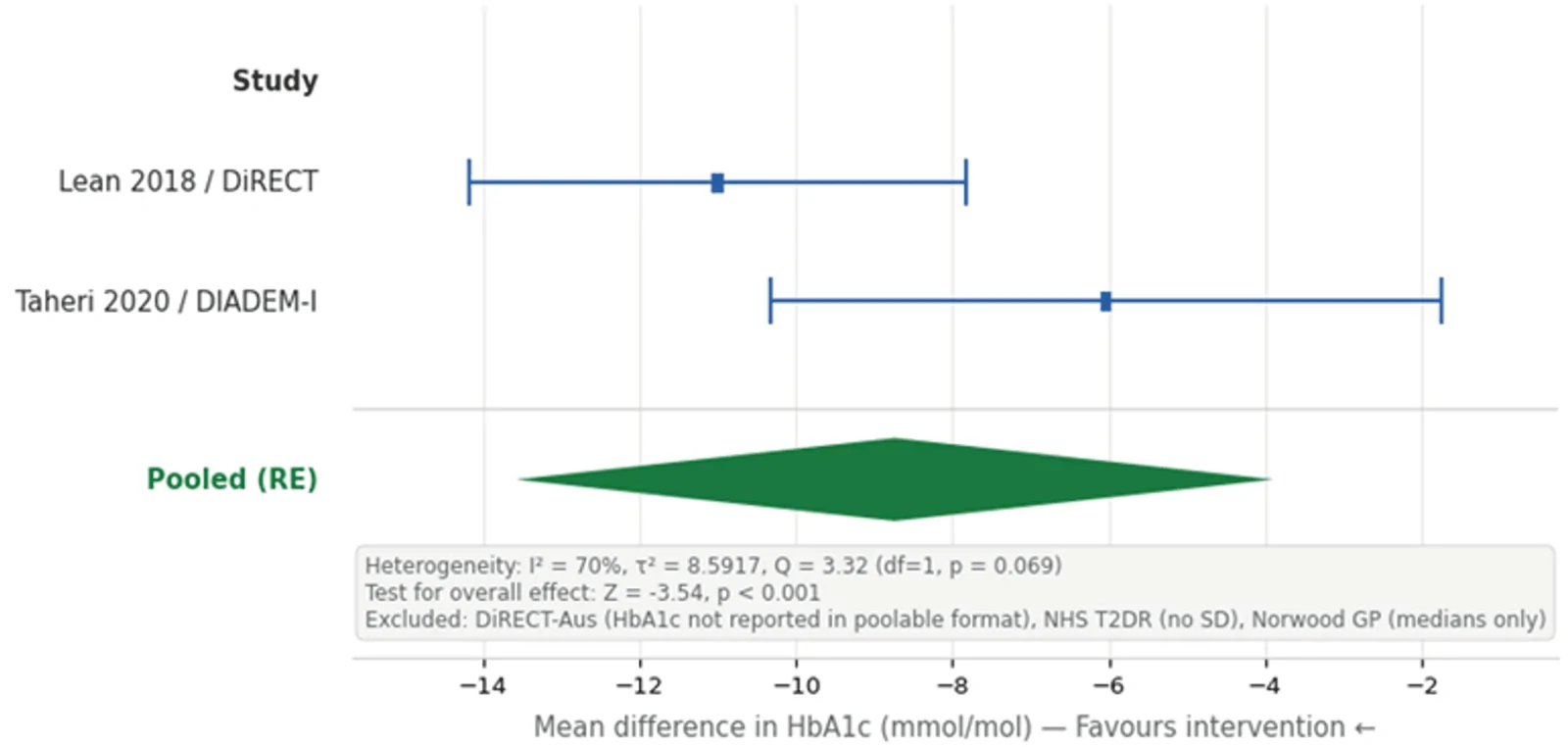
Forest plot of HbA1c change at 12 months Unadjusted between-group MDs in HbA1c change were pooled using a DerSimonian-Laird random-effects model [7]. Data were derived from Lean et al. (DiRECT) and Taheri et al. (DIADEM-I) [1,2]. Lifestyle interventions produced greater HbA1c reduction compared with usual care (pooled MD −8.74 mmol/mol, 95% CI −13.58 to −3.90), with moderate-to-substantial heterogeneity (I² = 69.8%). MD: mean difference, CI: confidence interval, HbA1c: glycated hemoglobin, DiRECT: Diabetes Remission Clinical Trial, DIADEM-I: Diabetes Intervention Acceleration Through Mobilization I

Meta-analyses were conducted separately according to evidence stream (Figure 2, Table 5; Figure 3, Table 6; Figure 4, Table 7; Figure 5, Table 8). Single-arm and implementation studies were not combined with comparative RCT estimates. At 12 months, DiRECT reported remission in 46% of intervention participants compared with 4% of controls, while DIADEM-I reported remission in 61% compared with 12% of controls [1,2]. The pooled RR for remission favored dietary intervention (RR 7.42, 95% CI 3.51-15.69).

**Table 5:** Study-level and pooled estimates of T2DM remission at 12 months T2DM: type 2 diabetes mellitus, RE: random-effects, I: intervention, C: control, RR: risk ratio, CI: confidence interval, DiRECT: Diabetes Remission Clinical Trial, DIADEM-I: Diabetes Intervention Acceleration Through Mobilization I

Study	Events (I vs C)	RR (95% CI)	Weight
Lean et al., 2018 [1] (DiRECT)	68/149 vs 6/149	11.33 (5.08-25.30)	44.80%
Taheri et al., 2020 [2] (DIADEM-I)	43/70 vs 9/77	5.26 (2.77-9.98)	55.20%
Pooled (RE)	111/219 vs 15/226	7.42 (3.51-15.69)	100%

**Table 6:** Study-level and pooled estimates of medication-free status at 12 months RE: random-effects, I: intervention, C: control, RR: risk ratio, CI: confidence interval, DiRECT: Diabetes Remission Clinical Trial, DIADEM-I: Diabetes Intervention Acceleration Through Mobilization I

Study	Events (I vs C)	RR (95% CI)	Weight
Lean et al., 2018 [1] (DiRECT)	109/148 vs 27/148	4.04 (2.83-5.75)	64.10%
Taheri et al. [2] (DIADEM-I)	64/68 vs 14/72	4.84 (3.01-7.77)	35.90%
Pooled (RE)	173/216 vs 41/220	4.31 (3.24-5.72)	100%

**Table 7:** Study-level and pooled estimates of weight change at 12 months RE: random-effects, I: intervention, C: control, MD: mean difference, CI: confidence interval, DiRECT: Diabetes Remission Clinical Trial, DIADEM-I: Diabetes Intervention Acceleration Through Mobilization I

Study	Participants (I vs C)	MD, kg (95% CI)	Weight
Lean et al., 2018 [1] (DiRECT)	137 vs 148	−9.00 (−10.47 to −7.53)	74.60%
Taheri et al., 2020 [2] (DIADEM-I)	70 vs 77	−8.00 (−10.51 to −5.49)	25.40%
Pooled (RE)	207 vs 225	−8.75 (−10.01 to −7.48)	100%

**Table 8:** Study-level and pooled estimates of HbA1c change at 12 months HbA1c: glycated hemoglobin, RE: random-effects, I: intervention, C: control, MD: mean difference, CI: confidence interval, DiRECT: Diabetes Remission Clinical Trial, DIADEM-I: Diabetes Intervention Acceleration Through Mobilization I

Study	Participants (I vs C)	MD, mmol/mol (95% CI)	Weight
Lean et al., 2018 [1] (DiRECT)	138 vs 148	−11.00 (−14.18 to −7.82)	54.40%
Taheri et al., 2020 [2] (DIADEM-I)	67 vs 75	−6.04 (−10.33 to −1.75)	45.60%
Pooled (RE)	205 vs 223	−8.74 (−13.58 to −3.90)	100%

Medication-free status was achieved in 74% of intervention participants versus 18% of controls in DiRECT and 94% versus 19% in DIADEM-I [1,2]. The pooled RR for medication-free status was 4.31 (95% CI 3.24-5.72). Weight loss was greater with intervention in both RCTs. Mean weight change was −10.0 kg versus −1.0 kg in DiRECT and −11.98 kg versus −3.98 kg in DIADEM-I [1,2]. The pooled unadjusted MD was −8.75 kg (95% CI −10.01 to −7.48). HbA1c reduction was also greater with intervention, with a pooled MD of −8.74 mmol/mol (95% CI −13.58 to −3.90), although heterogeneity was substantial.

STANDby was analyzed separately because of its short follow-up and proof-of-concept design. Remission occurred in 38% of intervention participants compared with none of the controls, with a mean weight difference of −6.3 kg and an HbA1c difference of −4.8 mmol/mol [3]. Single-arm implementation studies showed remission proportions ranging from 27% to 56%. DiRECT-Aus reported 56% remission at 12 months, NHS T2DR reported 27% using the primary denominator, Norwood GP reported 46% remission over a mean of 23 months, and STANDby observational follow-up reported 43% remission [3-6]. These findings were interpreted as implementation outcomes rather than comparative treatment effects (Table 9).

**Table 9:** Clinical outcomes across included lifestyle intervention studies Summary of remission, glucose-lowering medication deintensification, weight change, HbA1c outcomes, and serious adverse events across randomized, observational, and real-world implementation studies. Remission was defined according to individual study criteria, generally requiring HbA1c below 6.5% (48 mmol/mol) without glucose-lowering medication. Continuous outcomes are presented as MDs where available; studies reporting median values or without SD data were excluded from pooled MD analyses. CI: confidence interval, GLM: glucose-lowering medication, HbA1c: glycated hemoglobin, ITT: intention-to-treat, MD: mean difference, NS: not significant, OR: odds ratio, RR: risk ratio, SAE: serious adverse event, SD: standard deviation, TDR: total diet replacement, IQR: interquartile range, DiRECT: Diabetes Remission Clinical Trial, DIADEM-I: Diabetes Intervention Acceleration Through Mobilization I, DiRECT-Aus: Diabetes Remission Clinical Trial-Australia, NHS T2DR: National Health Service Type 2 Diabetes Remission

Study ID	N analysed	Follow-up	Remission, n/N (%)	Medication deintensification	Weight change	HbA1c change (mmol/mol)	Serious adverse events
Lean et al., 2018 [1] (DiRECT)	149/149	12 months	Intervention: 68/149 (46%) vs control: 6/149 (4%); OR 19.7 (95% CI 7.8-49.8)	Medication-free: 109/148 (74%) vs 27/148 (18%); mean change: −0.8 vs +0.2 medications	Intervention: −10.0 kg (SD 8.0) vs control: −1.0 kg (SD 3.7); unadjusted MD: −9.0 kg	Intervention: −9.6 (SD 15.4) vs control: +1.4 (SD 11.6); unadjusted MD: −11.0 mmol/mol	9 SAEs in 7 intervention participants (4%) vs 2 SAEs in 2 control participants (1%)
Taheri et al., 2020 [2] (DIADEM-I)	70/77	12 months	Intervention: 43/70 (61%) vs control: 9/77 (12%); OR 12.03 (95% CI 5.17-28.03). Normoglycaemia: 23/70 (33%) vs 3/77 (4%)	Medication-free: 64/68 (94%) vs 14/72 (19%); mean change: −1.38 vs +0.06 medications	Intervention: −11.98 kg (SD 9.46) vs control: −3.98 kg (SD 5.29); unadjusted MD: −8.00 kg; adjusted MD: −6.08 kg (95% CI −8.37 to −3.79)	Intervention: −9.50 (SD 11.31) vs control: −3.46 (SD 14.70); unadjusted MD: −6.04 mmol/mol; adjusted MD: −6.77 mmol/mol (95% CI −10.09 to −3.46)	5 SAEs in 4 control participants; none in intervention group
Sattar et al., 2023 [3] (STANDby, RCT phase)	46369	Post-TDR (~105 days)	5/13 (38%) vs 0/12 (0%); RR not calculable	Medication-free: 10/13 (77%) vs 3/12 (25%)	Intervention: −7.2 kg (SD 7.8) vs control: −0.9 kg (SD 1.2); unadjusted MD: −6.3 kg	Intervention: −5.5 (SD 11.0) vs control: −0.7 (SD 5.6); unadjusted MD: −4.8 mmol/mol (NS)	None reported
Sattar et al., 2023 [3] (STANDby, observational)	23	Median 105 days	10/23 (43%)	7/12 baseline medication users remained off diabetes medication after TDR	Mean −6.8 kg (95% CI −9.5 to −4.0)	Mean −7.2 mmol/mol (95% CI −12.4 to −2.0)	None reported
Hocking et al., 2024 [4] (DiRECT-Aus)	155 (ITT)	12 months	Primary: 86/155 (56%); consensus: 85/155 (55%)	95/110 (86%) not taking GLM within 60 days of 12-month completion	Mean −8.1% body weight (ITT); −9.3% among completers. SD not reported - excluded from pooled MD	Not reported in mean/SD format - excluded from pooled MD	2 SAEs: hospitalizations for hypotension (1 with hypoglycemia)
Valabhji et al., 2024 [5] (NHS T2DR)	1,710 (full-opportunity)	12 months	Primary: 190/710 (27%); completers: 145/450 (32%); conservative: 190/1,710 (11%)	Medication-free status embedded in remission definition	Mean −9.4 kg (95% CI −9.8 to −8.9); −10.3 kg among completers. SD not reported - excluded from pooled MD	Not reported in mean/SD format - excluded from pooled MD	Not reported
Unwin et al., 2020 [6] (Norwood GP)	128	Mean 23 months	59/128 (46%) drug-free remission	89/128 (70%) medication-free; 35% of previously medicated participants became medication-free; 4 stopped insulin	Median ~−8.3 kg. Median/IQR only - excluded from pooled MD	Median ~−17.5 mmol/mol (65.5 to 48). Median/IQR only - excluded from pooled MD	Not reported

Narrative Synthesis

Across studies, remission was defined as HbA1c below 48 mmol/mol without glucose-lowering medication. Intervention-based approaches consistently produced clinically meaningful weight loss, improved glycemic control, and reduced medication requirements [1-6]. Greater weight loss was generally associated with higher remission rates, although differences in study design, populations, and follow-up periods limited direct comparison.

Medication-free status was frequently more common than formal remission. This was observed in DiRECT, DIADEM-I, STANDby, DiRECT-Aus, and Norwood GP, although interpretation was limited by variation in definitions and follow-up duration [1-6].

Safety outcomes were generally favorable, with serious adverse events uncommon. Reported events included isolated episodes of hypotension and hypoglycemia in DiRECT-Aus and serious adverse events occurring mainly among control participants in DIADEM-I [2,4].

The studies demonstrated that remission-focused dietary interventions could be delivered through primary care. DiRECT showed that trained primary care staff could deliver TDR with structured support, while NHS T2DR demonstrated national-scale implementation with challenges related to program completion and outcome ascertainment [1,5].

Certainty of Evidence

For the comparative RCT evidence, certainty was moderate for remission, medication-free status, and weight change because of study limitations related to open-label design and limited trial numbers [1,2]. Certainty for HbA1c outcomes was lower due to heterogeneity between trials. Evidence from implementation studies was considered very low certainty because of the absence of comparator groups, risk of bias, and variability in reported outcomes [4-6].

Discussion

This systematic review demonstrates that primary care-linked lifestyle interventions, particularly TDR-based programs, can substantially increase the likelihood of T2DM remission and glucose-lowering medication deintensification in selected adults. Findings from the two 12-month RCTs, DiRECT and DIADEM-I, consistently showed higher remission rates, greater medication-free status, and greater weight loss than usual care, supporting the effectiveness of intensive dietary weight-management approaches when delivered with structured clinical support [1,2].

The observed benefits appear to be closely associated with substantial and sustained weight loss. Previous mechanistic work has demonstrated that reducing excess body weight can decrease ectopic fat accumulation in the liver and pancreas, improving insulin sensitivity and beta-cell function in individuals with sufficient metabolic reserve [1,2,5]. The included studies support this relationship, as greater weight reduction was generally accompanied by improved glycemic outcomes and reduced medication requirements [1-6]. However, remission was not achieved universally, highlighting the influence of individual factors such as diabetes duration, baseline characteristics, and beta-cell functional reserve [1,2,5].

Beyond efficacy, this review highlights the importance of implementation within routine primary care. DiRECT and DIADEM-I demonstrated that remission-focused interventions can be integrated into primary care-linked pathways with structured medication withdrawal and follow-up procedures [1,2]. Real-world evidence from DiRECT-Aus supports implementation in routine primary care, while NHS T2DR demonstrates the potential for delivery at national scale. However, remission rates in implementation settings should be interpreted as service outcomes rather than direct estimates of treatment effectiveness because of the absence of comparator groups and greater methodological limitations [4,5].

Medication deintensification represents an important clinical outcome alongside remission because many participants achieved medication-free status, even when complete remission was not reached [1-6]. Safe reduction of glucose-lowering therapy requires appropriate monitoring and predefined protocols, as demonstrated across the included studies [1-5]. Lower-carbohydrate dietary approaches delivered through primary care may also contribute to improved glycemic control and reduced medication burden. However, the evidence base remains less robust than that for TDR interventions [6].

Overall, the evidence supports incorporating structured lifestyle remission pathways into primary care for appropriately selected adults with T2DM. Future research should focus on improving long-term maintenance of remission, identifying individuals most likely to benefit, and evaluating the effectiveness of these interventions across broader and more diverse healthcare settings [1-6].

Limitations

The evidence base was limited by the small number of 12-month comparative RCTs, restricting precision and generalisability. All RCTs were open-label, and potential performance and detection bias could not be excluded, although objective metabolic outcomes may reduce this concern. Some studies had additional methodological limitations, including cluster randomization, small sample sizes, and disruptions to follow-up. Single-arm and implementation studies lacked comparator groups and therefore could not establish causal effects. Variations in remission definitions, medication-free duration criteria, and outcome reporting limited data pooling. Publication bias could not be formally assessed because of the small number of studies. Evidence on long-term remission durability, relapse, cost-effectiveness, cardiovascular outcomes, and mortality remains limited.

## Conclusions

Primary care-linked lifestyle interventions, particularly structured TDR-based pathways, represent a promising approach for achieving T2DM remission and reducing reliance on glucose-lowering medication in appropriately selected adults. The effectiveness of these interventions appears closely linked to sustained weight loss, supported by structured dietary support, medication management, and ongoing follow-up. Translating research findings into routine primary care requires attention to implementation factors, including intervention accessibility, participant support, monitoring processes, and long-term maintenance strategies. Future research should prioritize standardized outcome definitions, broader population representation, and longer-term evaluation of remission durability and clinical outcomes. Integrating structured lifestyle remission pathways into primary care may provide an important component of comprehensive T2DM management.

## References

[1] Lean ME, Leslie WS, Barnes AC (2018). Primary care-led weight management for remission of type 2 diabetes (DiRECT): an open-label, cluster-randomised trial. Lancet.

[2] Taheri S, Zaghloul H, Chagoury O (2020). Effect of intensive lifestyle intervention on bodyweight and glycaemia in early type 2 diabetes (DIADEM-I): an open-label, parallel-group, randomised controlled trial. Lancet Diabetes Endocrinol.

[3] Sattar N, Welsh P, Leslie WS (2023). Dietary weight-management for type 2 diabetes remissions in South Asians: the South Asian diabetes remission randomised trial for proof-of-concept and feasibility (STANDby). Lancet Reg Health Southeast Asia.

[4] Hocking SL, Markovic TP, Lee CM, Picone TJ, Gudorf KE, Colagiuri S (2024). Intensive lifestyle intervention for remission of early type 2 diabetes in primary care in Australia: DiRECT-Aus. Diabetes Care.

[5] Valabhji J, Gorton T, Barron E (2024). Early findings from the NHS type 2 diabetes path to remission programme: a prospective evaluation of real-world implementation. Lancet Diabetes Endocrinol.

[6] Unwin D, Khalid AA, Unwin J (2020). Insights from a general practice service evaluation supporting a lower carbohydrate diet in patients with type 2 diabetes mellitus and prediabetes: a secondary analysis of routine clinic data including HbA1c, weight and prescribing over 6 years. BMJ Nutr Prev Health.

[7] DerSimonian R, Laird N (1986). Meta-analysis in clinical trials. Control Clin Trials.

[8] Goldenberg JZ, Day A, Brinkworth GD (2021). Efficacy and safety of low and very low carbohydrate diets for type 2 diabetes remission: systematic review and meta-analysis of published and unpublished randomized trial data. BMJ.

[9] Taylor R, Al-Mrabeh A, Zhyzhneuskaya S (2018). Remission of human type 2 diabetes requires decrease in liver and pancreas fat content but is dependent upon capacity for β cell recovery. Cell Metab.

[10] Page MJ, McKenzie JE, Bossuyt PM (2021). The PRISMA 2020 statement: an updated guideline for reporting systematic reviews. Rev Esp Cardiol (Engl Ed).

[11] Crocker TF, Lam N, Jordão M (2023). Risk-of-bias assessment using Cochrane's revised tool for randomized trials (RoB 2) was useful but challenging and resource-intensive: observations from a systematic review. J Clin Epidemiol.

[12] Hilton M (2024). JBI critical appraisal checklist for systematic reviews and research syntheses. J Can Health Libr Assoc.

[13] Freeman MF, Tukey JW (1950). Transformations related to the angular and the square root. Ann Math Stat.

[14] Guyatt GH, Oxman AD, Schünemann HJ, Tugwell P, Knottnerus A (2011). GRADE guidelines: a new series of articles in the Journal of Clinical Epidemiology. J Clin Epidemiol.

